# Growth speed of large brain metastases between diagnostic and radiosurgical planning MRI and predictors of rapid tumor growth

**DOI:** 10.1007/s11604-023-01524-w

**Published:** 2024-01-12

**Authors:** Shoko Takata, Kazuhisa Kosen, Akira Matsumoto, Motoko Tanabe, Takayoshi Itaya, Yoshiki Asayama

**Affiliations:** 1 Department of Radiology, Faculty of Medicine, Oita University, 1-1 Idaigaoka, Hasama-machi, Yufu, Oita 879-5593 Japan; 2 Department of Radiology, Oita Prefectural Hospital, 2-8-1, Bunyo, Oita 870-8511 Japan; 3 Keiwakai Oka Hospital, 3-7-11 Nishitsurusaki, Oita, 870-0105 Japan; 4 Department of Radiation Therapy, Central Japan International Medical Center, 1-1 Kenkonomachi, Minokamo, Gifu 505-8510 Japan

**Keywords:** Brain metastases, Growth rate, Percentage growth rate, Fractionated stereotactic radiation surgery, Predictors

## Abstract

**Purpose:**

We aimed to assess volumetric changes of large brain metastases (≥ 2 cm) between their diagnosis and planning for treatment with fractionated stereotactic radiation surgery (fSRS). Predictors of rapid tumor growth were also analyzed.

**Materials and methods:**

One hundred nine patients harboring 126 large brain metastases were retrospectively evaluated. Tumor characteristics were evaluated on diagnostic magnetic resonance imaging (dMRI) and MRI performed when planning fSRS (pMRI). Average tumor growth rate and percentage growth rate were calculated. Predictors of rapid growth (percentage growth rate > 5%) were determined using multivariate logistic regression.

**Results:**

Both tumor diameter and volume were significantly larger on pMRI than on dMRI (*P* < 0.001). Median tumor percentage growth rate was 2.6% (range, − 10.8–43.3%). Eighty-eight tumors (70%) were slow-growing (percentage growth rate < 5%) and 38 (30%) grew rapidly (percentage growth rate ≥ 5%). Major peritumoral edema and no steroids were predictors of rapid tumor growth.

**Conclusion:**

Large brain metastases can grow considerably between the time of diagnosis and the time of fSRS treatment planning. We recommend the time between dMRI and fSRS treatment initiation be as short as possible.

## Introduction

Brain metastases are a substantial contributor to overall cancer mortality and poor prognosis in patients with advanced-stage cancer [1]. Conventional whole-brain radiotherapy is an important and widely used brain metastasis treatment modality, particularly in patients with multiple lesions. However, large-field brain radiotherapy is associated with neuropsychological sequelae and deterioration in quality of life [1–3]. Therefore, single-fraction stereotactic radiosurgery (sfSRS) has become the preferred treatment for patients with one or several brain metastases because of its superior toxicity profile and high rate of local control [4].

Patients with large brain metastases are more likely to experience severe radiation toxicity and are consequently treated using a lower radiation dose, which frequently results in local failure [5, 6]. For brain metastases ≥ 2 cm in size, fractionated SRS (fSRS) is associated with a higher rate of local control and lower rate of radiation toxicity than those with sfSRS [3, 5]. Given that SRS uses high biologically equivalent doses, current recurrence patterns after SRS may be driven by inaccuracies in target delineation rather than by insufficient dose [3].

To ensure that the clinical target volume is irradiated with the prescribed dose, a margin is added to the clinical target volume to account for geometric uncertainty and patient motion. The volume with the added margin is defined as the planning target volume (PTV). For SRS, the PTV margin should be as small as possible to reduce the risk of radiation toxicity. If the tumor grows beyond the PTV margin between SRS-planning magnetic resonance imaging (MRI) and delivery of treatment, the tumor may not receive an adequate radiation dose. Although previous studies have reported growth rates of small brain metastases before sfSRS planning [7–9], to the best of our knowledge, none have examined tumor growth speed of large (≥ 2 cm) brain metastases before fSRS planning. Therefore, we aimed to examine the growth speed of large brain metastases between diagnostic and radiosurgical planning MRI and investigate the predictors of rapid tumor growth.

## Materials and methods

### Patients

**Fig. 1:**
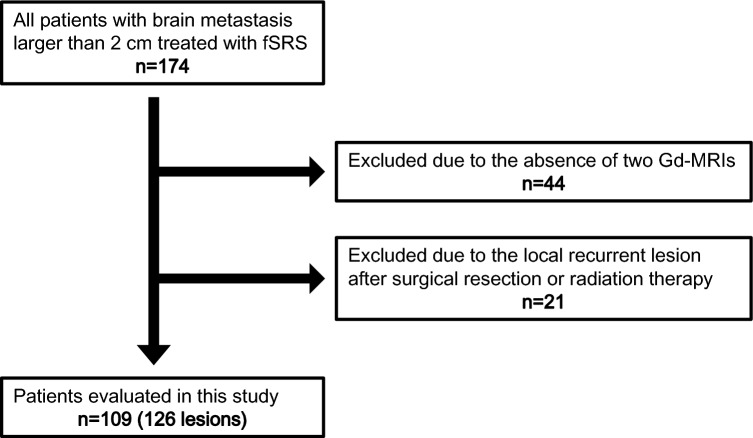
Study flowchart. *fSRS* fractionated stereotactic radiation surgery; *Gd-MRI* gadolinium-enhanced magnetic resonance imaging

This study was approved by the Ethics Committee of Oita University Faculty of Medicine (protocol number 2218). The requirement for informed written consent was waived. We retrospectively reviewed the medical records of 174 patients with brain metastases > 2 cm in size who were treated with fSRS (CyberKnife; Accuray, Inc., Sunnyvale, CA, USA) at Oka Hospital in Oita, Japan, from November 2016 to December 2021. Twenty-one patients with recurrent metastases after surgical resection or radiation therapy were excluded. We also excluded 44 in whom both diagnostic and radiosurgical planning MRI data were unavailable. Therefore, data for 109 patients harboring 126 brain metastases were included for analysis (Fig. 1).

### MRI

Diagnostic MRI (dMRI) protocols varied between patients, because the patients were referred from numerous hospitals. Planning MRI (pMRI) was performed in all patients at Oka Hospital with a 1.5-T system using two protocols. Magnetization-prepared rapid gradient-echo imaging (TR/TE = 1730/4.3 ms, flip angle = 15°, resolution = 0.9 × 0.9 × 1.0 mm) and volumetric interpolated brain examination imaging (TR/TE = 4.14/1.58 ms, flip angle = 12°, resolution = 0.9 × 0.9 × 1.0 mm) were selected on the basis of the time the patient could remain at rest. Axial contrast-enhanced T1-weighted images were acquired in planes parallel to the bicommissural line 5–15 min after injection of standard-dose (0.1 mmol/kg) or double-dose (0.2 mmol/kg) gadolinium. The slice thickness was 1.0 mm. A thermoplastic mask was used for patient immobilization.

### Clinical data and imaging evaluation

**Fig. 2:**
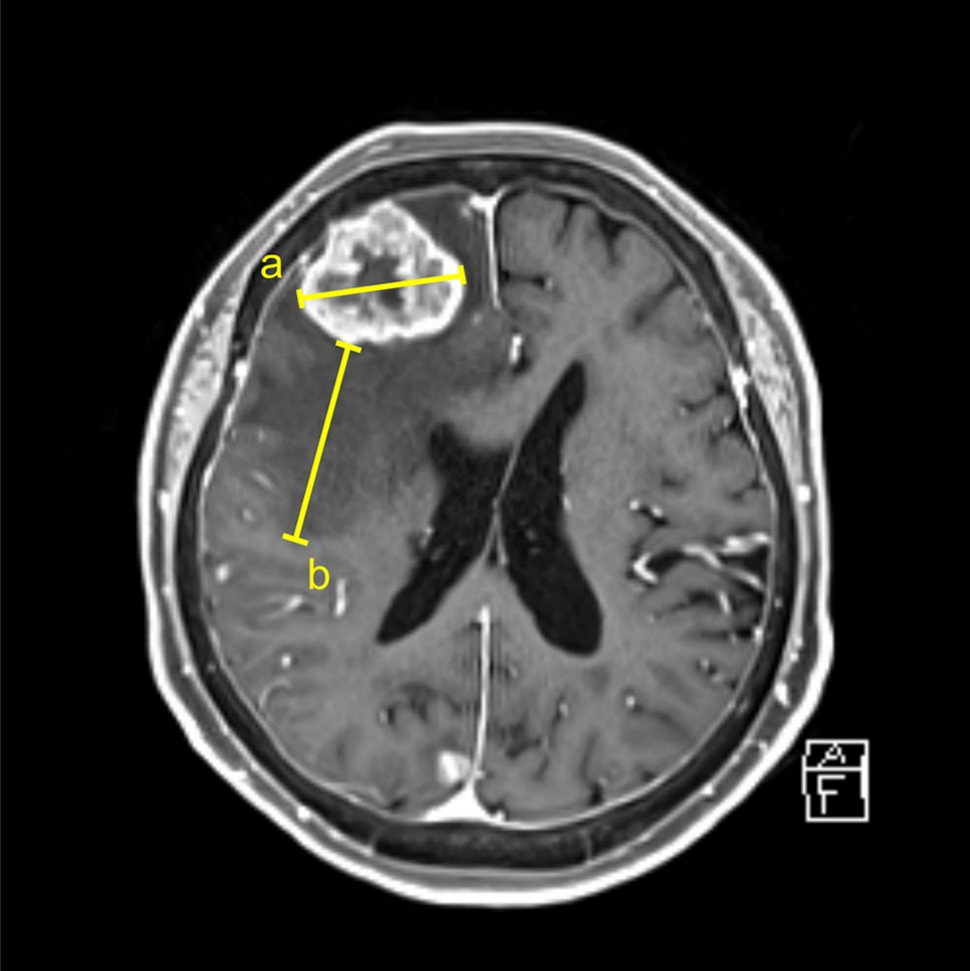
**a** Maximum tumor diameter was measured on axial contrast-enhanced T1-weighted imaging. **b** The extent of peritumoral edema was measured on the same slice as the maximum length from the tumor margin to the edge of the edema

Patient data, namely age, sex, primary site, steroid use, and time between dMRI and pMRI, were recorded. The diagnoses of the primary tumor and brain metastases were based on imaging and pathological data. Tumor characteristics (solid vs cystic or hemorrhagic) and degree of peritumoral edema were recorded on dMRI, while tumor diameter and volume were measured on both dMRI and pMRI contrast-enhanced T1-weighted images. On the axial slice showing the maximum tumor diameter, maximum tumor diameter and degree of peritumoral edema were measured. The latter was measured as the maximum length from the tumor margin to the edge of the edema (Fig. 2). Length rather than volume of the edema was used, because the former is easier to measure. The ratio of the edema and tumor measurements was calculated as the edema/tumor ratio. Tumors were delineated on each slice, and tumor volume was calculated using Aquarius iNtuition Edition version 4.4.11 (TeraRecon, Inc., Foster City, CA, USA). Average growth rate was calculated by dividing the difference in tumor volume by the time interval. Relative change in tumor volume was expressed as percentage growth rate.

### Statistical analysis

Statistical analyses were performed using SPSS software version 24.0 (IBM Corp., Armonk, NY, USA). Tumor diameter and volume were compared using the Wilcoxon rank sum test. The Kruskal–Wallis test was used to assess intergroup differences. The Chi-square test and Mann–Whitney *U* test were used to compare characteristics between different percentage growth rates. Odds ratios (ORs) with 95% confidence intervals (CIs) were calculated using logistic regression to determine predictors of rapid tumor growth. All variables that were significant in the univariate analysis (*P* < 0.1) were entered into a multivariate analysis, in which *P* < 0.05 was considered significant.

## Results

### Patient characteristics and imaging features

Patient and pMRI characteristics are shown in Table 1. Median patient age was 68 years (range, 23–90). Sixty-five patients were men and 44 were women. The most common primary tumor was lung cancer (*n* = 64, 56%), with the following histopathologic types: adenocarcinoma (*n* = 29), squamous cell carcinoma (*n* = 7), small cell carcinoma (*n* = 9), other (*n* = 7), and unknown (*n* = 12). Breast cancer was next in frequency (*n* = 10, 9%), with no detailed histopathologic type. Others comprised stomach (*n* = 6, 6%), colon (*n* = 4, 4%), esophageal (*n* = 4, 4%), and ovarian (*n* = 4, 4%) cancers, melanoma (*n* = 2), sarcoma (*n* = 2), cancer of unknown primary (*n* = 1), and renal (*n* = 2), pancreatic (*n* = 2), thymic (*n* = 1), endometrial (*n* = 2), prostate (*n* = 2), thyroid (*n* = 1), testicular (*n* = 1), and oral cancers (*n* = 1). For primary site tumors, excluding lung and breast tumors, the histologic type of unknown lesions (*n* = 23) was more common than known lesions (*n* = 12). Forty-five (41%) patients were taking a steroid on the day of pMRI. Among the 126 brain metastases, 44 (36%) were solid and 82 (64%) were cystic or hemorrhagic. The median peritumoral edema extension on dMRI was 2.1 cm, and the range was 0–5.9 cm. Minor edema (edema/tumor ratio < 1) was observed in 84 (67%) patients; 42 (33%) demonstrated major edema (edema/tumor ratio > 1).

**Table 1:** Patient characteristics and imaging features

Characteristics	Patients (*n* = 109)
Age (years)	68 [23, 90]
Sex (male/female)	65/44
Primary site (lung/breast/other)	64/10/35
Taking steroid (yes/no)	45/64
dMRI to pMRI (days)	12 [1, 63]

Imaging features	Lesions (*n *= 126)
Characteristics (solid/cystic or hemorrhagic)	44/82
Edema (cm)	2.1 [0, 5.9]
Growth rate (cm^3^/day)	0.2 [−1.3, 4.8]
Percentage growth rate (%)	2.6 [−10.8, 43.3]

*dMRI* diagnostic magnetic resonance imaging; *pMRI* planning magnetic resonance imaging

Data shown are medians with range or numbers

### Size change in tumors

Both tumor diameter and volume were significantly larger on pMRI than dMRI (*P* < 0.001). Median maximum tumor diameter was 2.7 cm (range, 1.4–6.8) on dMRI and 3.0 cm (range, 2.0–7.6) on pMRI. Median tumor volume was 7.9 cm^3^ (range, 0.9–96) on dMRI and 9.6 cm^3^ (range, 2.1–122) on pMRI. The median average growth rate overall was 0.21 cm^3^/day (range, − 1.3–4.8). The median average growth rate was 0.20 cm^3^/day for lung cancer (most common tumor) and 0.14 cm^3^/day for breast cancer (second most common); the rate for other tumors was 0.29 cm^3^/day (*P* = 0.011). The median tumor percentage growth rate overall was 2.6% (range, − 10.8%–43.3%); the rates for lung cancer, breast cancer, and other tumors were 2.3%, 0.8%, and 4.4%, respectively (*P* = 0.005).

We defined rapid growth as a growth rate of ≥ 5%. Assuming a linear increase in tumor volume in a perfectly spherical shape, this means that a 2-cm tumor would grow beyond a 1-mm margin within 7 days, and a 4-cm tumor would grow beyond the margin within 4 days. Fourteen tumors (11%) decreased in size, 74 (59%) were slow-growing (percentage growth rate < 5%), and 38 (30%) grew rapidly (percentage growth rate ≥ 5%). Among the rapidly growing tumors, the percentage growth rate was 10–20% in seven and > 20% in four.

### Predictors of rapid tumor growth

Table 2 shows the results of the univariate and multivariate analyses of the clinical factors influencing rapid tumor growth. Sex (*P* = 0.047), taking steroids (*P* = 0.041), and edema ratio (*P* = 0.077) were associated with rapid growth in the univariate analysis. Multivariate logistic regression demonstrated that not taking steroids (OR 2.62; 95% CI 1.11–6.21; *P* = 0.028) and major edema (OR 2.37; 95% CI 1.02–5.50; *P* = 0.045) were predictors of rapid growth.

**Table 2:** Analysis of factors associated with rapid tumor growth

	Univariate analysis			Multivariate analysis		
	Odds ratio	95% CI	*P*	Odds ratio	95% CI	*P*
Sex						
Male	2.33	1.01–5.38	0.047	2.16	0.91–5.10	0.080
Female	1			1		
Age (years)						
< 60	1.08	0.38–3.10	0.884			
≥ 60	1					
Primary site						
Lung	1					
Breast	0.23	0.03–1.89	0.229			
Others	1.68	0.75–3.78	1.683			
Taking steroid						
Yes	1			1		
No	2.35	1.04–5.31	0.041	2.62	1.11–6.21	0.028
Characteristics						
Solid	1.13	0.51–2.49	0.766			
Cystic or hemorrhagic	1					
Edema ratio						
< 1	1			1		
≥ 1	2.04	0.93–4.49	0.077	2.37	1.02–5.50	0.045
Tumor diameter						
< 3 cm	1					
≥ 3 cm	0.60	0.27–1.31	0.199			
Tumor volume						
< 8 cm^3^	1					
≥ 8 cm^3^	0.61	0.28–1.31	0.606			

*CI* confidence interval

## Discussion

This study demonstrated that large brain metastases can grow remarkably in the short period of time between dMRI and pMRI. Such measurable changes in tumor volume may affect the actual dose delivered to the tumor margins, because the dose drops sharply around the target in SRS. Furthermore, inaccurate delineation of the target dose may lead to recurrence after SRS [3]. Almost 90% of the tumors in our study grew and almost one-third grew rapidly (percentage growth rate > 5%) between dMRI and pMRI. Amazingly, a tumor with a 43% growth rate, which was the highest rate we observed, would grow beyond a 1-mm margin in only 1 day. The smallest percentage growth rate we observed was − 14%: a tumor with this percentage growth rate would shrink within 1 mm of the tumor margin in 2 days.

Our study of large brain metastases showed that tumor volume increases in a short period of time. This finding is similar to those in previous reports measuring brain metastases of various sizes in non-small cell lung cancer and melanoma, which showed that tumor progression between dMRI and pMRI was observed in 82% of cases [7]. However, our average growth rate of 0.21 cm^3^/day appears to be considerably higher than previously reported rates. In a paper measuring the natural growth rates of brain metastases from breast and lung cancer, mean initial tumor volume was 4.45 cm^3^, and the average growth velocity was 0.034 cm^3^/day [8]. In another study of brain metastases from melanoma, breast cancer, and others, initial mean volume was 1.08 mL, and the mean absolute growth rate was 0.02 mL/day [9]. Recently, a new mathematical model for the volume growth of brain metastases has been reported. Tumors often obey the so-called scaling laws that relate an observable quantity to a measure of the size of the system. The scaling exponent for brain metastases is superlinear, which implies the potential for explosive volumetric growth [10]. The much larger growth rate reported in our study may also be related to the initially larger brain metastasis volume.

The rate of tumor growth appears to vary depending on the primary site. In our study, the growth rates for lung, breast, and other tumors were 0.20, 0.14, and 0.29 cm^3^/day, and the median tumor percentage growth rates were 2.3%, 0.8%, and 4.4%, respectively. A previous report indicated that melanoma brain metastases may grow faster than metastases from other primary tumors [7, 9]. In the present study, we were unable to evaluate differences in growth rates by pathology owing to an insufficient number of cases per pathology, and further accumulation of cases is desirable.

We also found that major peritumoral edema and no steroids were predictors of rapid tumor growth. Peritumoral edema is clinically important as it causes symptoms. Peritumoral edema is mediated by blood–brain barrier breakdown and is vasogenic in nature. Vascular endothelial growth factor and other inflammatory brain tumor products are also involved [11]. Major peritumoral edema (≥ 10 mm) in non-small cell lung cancer brain metastasis appears to be a predictor of worse response to SRS [12]. To our knowledge, no studies have examined the association between peritumoral edema and tumor growth rate in large brain metastases. Using the peritumoral edema ratio, an easily measurable radiological feature, it might be possible to predict rapid tumor growth rate in large brain metastases, and this ratio may be important in determining treatment strategies. However, these data in our study were not evaluated owing to an insufficient number of cases per histopathology. The extent of peritumoral edema varies according to the primary tumor [13], and more data are needed.

We found that steroid use was an independent predictor of slow growth. Steroids are recommended in patients with symptomatic brain metastases to provide temporary relief of symptoms related to increased intracranial pressure and edema by decreasing the permeability of tumor capillaries [14, 15]. For tumor cells, there is experimental and clinical evidence that steroids have direct effects on tumor cell proliferation and apoptosis [15]. Tumor shrinkage has also been observed in brain metastases [16]. Our finding may be explained by the effect of steroid administration on brain metastases. However, the molecular mechanisms underlying the effects of steroids on tumor cell proliferation are still poorly understood [15]. Although steroid use appeared to be a predictor of slow growth in the current study, it should be noted that we did not evaluate shape changes and tumor displacement by anti-edematous changes.

In this study, we assessed volumetric changes of large brain metastases between dMRI and pMRI. Several studies have described changes in brain metastases after pMRI. In one study, measurable changes that required a change in SRS treatment occurred in 41% of patients with a treatment-to-planning interval of 7 days and in 78% of patients with an interval > 7 days [17]. In another study, metastasis growth was associated with time between pMRI and treatment MRI and metastasis size [9]. Kubo et al. reported that treatment plan modification was required for over half of the tumors in a study that evaluated tumor size, displacement, and shape changes during the treatment period [18]. Thus, rapid and complex changes may continue to occur after pMRI. However, clinically, it is impractical to perform multiple MRI studies for all patients throughout the planning and treatment process. Therefore, clinicians should strive to shorten the period between dMRI and treatment initiation, and the need for this may be especially pronounced for large tumors. Major edema on dMRI and no steroid therapy is also an important consideration. Individualized treatment may be possible if there is a need for changes in margin settings, repeat MRIs, and treatment modifications.

This study had several limitations, including the retrospective design and that the study was conducted in a single center. The hospital where this study was conducted specializes in stereotactic radiotherapy, and the information we investigated was provided only by the referring hospitals. Notably, dMRI conditions varied from patient to patient, and pathology data were missing in 40% of the patients. The time between dMRI and pMRI ranged from 1 to 63 days, which may have been influenced by the judgment of the attending physicians when considering symptoms and previous treatment, as well as the medical system of the referring hospital.

In conclusion, large brain metastases can grow considerably in the period between MRI diagnosis and fSRS treatment planning. We recommend the time between dMRI and fSRS treatment initiation be as short as possible. Major peritumoral edema on dMRI and no steroids were predictors of rapid tumor growth.
